# Consumption: How to Prevent It and How to Live with It

**Published:** 1892-12

**Authors:** 


*Consumption: How to Prevent it and How to Live with it.* By N. S. DAVIS, Jun., A.M., M.D. Pp. viii., 143. Philadelphia: F. A. DAVIS. 1891.

In the treatment of Consumption, Dr. Davis has found it advantageous to take his patients, as it were, into confidence, by preparing a series of hygienic rules in which are explained the necessity and effect of their strict observance. This book is an expansion of these rules, and the matter contained is judiciously chosen and carefully stated, with a due appreciation of the extent of knowledge necessary to enable patients to help, but not to "doctor," themselves. Following a short description of the nature of Consumption and the influence of predisposition, is a useful chapter on "Prevention"; then in turn the influence of general hygienic conditions, pure air, climate, clothing, and exercise, is ably discussed.

The book is a successful attempt to popularise useful knowledge.

